# Discovering common pathogenetic processes between COVID-19 and sepsis by bioinformatics and system biology approach

**DOI:** 10.3389/fimmu.2022.975848

**Published:** 2022-08-31

**Authors:** Lu Lu, Le-Ping Liu, Rong Gui, Hang Dong, Yan-Rong Su, Xiong-Hui Zhou, Feng-Xia Liu

**Affiliations:** ^1^ Department of Blood Transfusion, The Third Xiangya Hospital of Central South University, Changsha, China; ^2^ Department of Pediatrics, The Third Xiangya Hospital, Central South University, Changsha, China; ^3^ Department of Laboratory Medicine, The Third Xiangya Hospital of Central South University, Changsha, China

**Keywords:** COVID-19, sepsis, differentially expressed gene (DEG), functional enrichment, gene ontology, protein–protein interaction (PPI), hub gene, drug molecule

## Abstract

Corona Virus Disease 2019 (COVID-19), an acute respiratory infectious disease caused by severe acute respiratory syndrome coronavirus-2 (SARS-CoV-2), has spread rapidly worldwide, resulting in a pandemic with a high mortality rate. In clinical practice, we have noted that many critically ill or critically ill patients with COVID-19 present with typical sepsis-related clinical manifestations, including multiple organ dysfunction syndrome, coagulopathy, and septic shock. In addition, it has been demonstrated that severe COVID-19 has some pathological similarities with sepsis, such as cytokine storm, hypercoagulable state after blood balance is disrupted and neutrophil dysfunction. Considering the parallels between COVID-19 and non-SARS-CoV-2 induced sepsis (hereafter referred to as sepsis), the aim of this study was to analyze the underlying molecular mechanisms between these two diseases by bioinformatics and a systems biology approach, providing new insights into the pathogenesis of COVID-19 and the development of new treatments. Specifically, the gene expression profiles of COVID-19 and sepsis patients were obtained from the Gene Expression Omnibus (GEO) database and compared to extract common differentially expressed genes (DEGs). Subsequently, common DEGs were used to investigate the genetic links between COVID-19 and sepsis. Based on enrichment analysis of common DEGs, many pathways closely related to inflammatory response were observed, such as Cytokine-cytokine receptor interaction pathway and NF-kappa B signaling pathway. In addition, protein-protein interaction networks and gene regulatory networks of common DEGs were constructed, and the analysis results showed that *ITGAM* may be a potential key biomarker base on regulatory analysis. Furthermore, a disease diagnostic model and risk prediction nomogram for COVID-19 were constructed using machine learning methods. Finally, potential therapeutic agents, including progesterone and emetine, were screened through drug-protein interaction networks and molecular docking simulations. We hope to provide new strategies for future research and treatment related to COVID-19 by elucidating the pathogenesis and genetic mechanisms between COVID-19 and sepsis.

## Introduction

The novel coronavirus, SARS-CoV-2, is the causative agent of an atypical respiratory disease that has caused a global pandemic since 2019. The World Health Organization defines the infectious disease caused by the virus as Corona Virus Disease 2019 (COVID-19) (1). Since the pandemic, the new coronavirus has undergone a variety of mutations, and it has now mutated to Omicron BA.4 and BA.5, which has a strong immune evasion ability. Data showed that as of 31 December 2021, over 287 million cases had occurred worldwide, including more than 5.4 million deaths (2). More than 80% of COVID-19 patients have mild disease, but the incidence of severe or high-risk disease varies among patient populations (3). Literature suggests that critical illness including respiratory failure, multi-organ damage or shock can occur in up to 5% of patients (2). Severe COVID-19 is often pathologically manifested by pulmonary and extrapulmonary organ dysfunction. Studies have shown that the lung is the organ most severely affected by SARS-CoV-2, manifesting as diffuse alveolar damage, exudation, and interstitial fibrosis, accompanied by a large number of immune cell infiltration and inflammatory factor expression (3–5). Extrapulmonary organs have different degrees of tissue damage and inflammatory response, manifested as multiple organ dysfunction and systemic inflammatory response (6). In terms of clinical symptoms, most severe COVID-19 patients eventually develop typical septic shock manifestations, including cold limbs, microcirculatory dysfunction, weak peripheral pulse, oxidative stress injury, and cytokine storm (7). In addition, in clinical care, the latest COVID-19 treatment guidelines, “surviving sepsis campaign”, have been adopted as treatment guidelines for critically ill patients (8). All in all, both in terms of clinical diagnosis and treatment, severe COVID-19 and sepsis have similarities, and the two can learn from each other.

Sepsis is a systemic inflammatory response syndrome (SIRS) caused by a variety of factors, including infection, trauma and surgery, and its mortality and morbidity are extremely high (5). Uncontrolled inflammation and overproduction of Reactive Oxygen and Nitrogen Species (RONS) are the hallmarks of sepsis, which in turn cause cell and tissue destruction, immune system dysfunction, and marked hemopathology, ultimately leading to multiple organ failure syndrome Signs (MODS) (9–13). Part of the viral pneumonia caused by SARS-CoV-2 is a fulminant disease with similar manifestations to sepsis (14). Considering the similarities between COVID-19 and non-SARS-CoV-2 induced sepsis, it is necessary to understand the biological links and potential molecular mechanisms between the two to provide new insights into the pathogenesis of COVID-19 and to search for potential therapeutic agents for patients with COVID-19 or patients with COVID-19 secondary to sepsis.

With the development of science and technology, biology and computer technology are becoming more and more closely integrated. Bioinformatics is a discipline that uses computer algorithms to effectively analyze biological data, enabling a systematic approach to understanding the developmental process of organisms, classifying organisms, studying biomarkers of diseases, etc (15). Machine learning is a kind of algorithm of artificial intelligence, which can explore potential laws in massive data. It has high accuracy and has emerged in medical research and medical development (16). In recent years, machine learning and bioinformatics analysis have played an important role in medical research and application.

**Figure 1:**
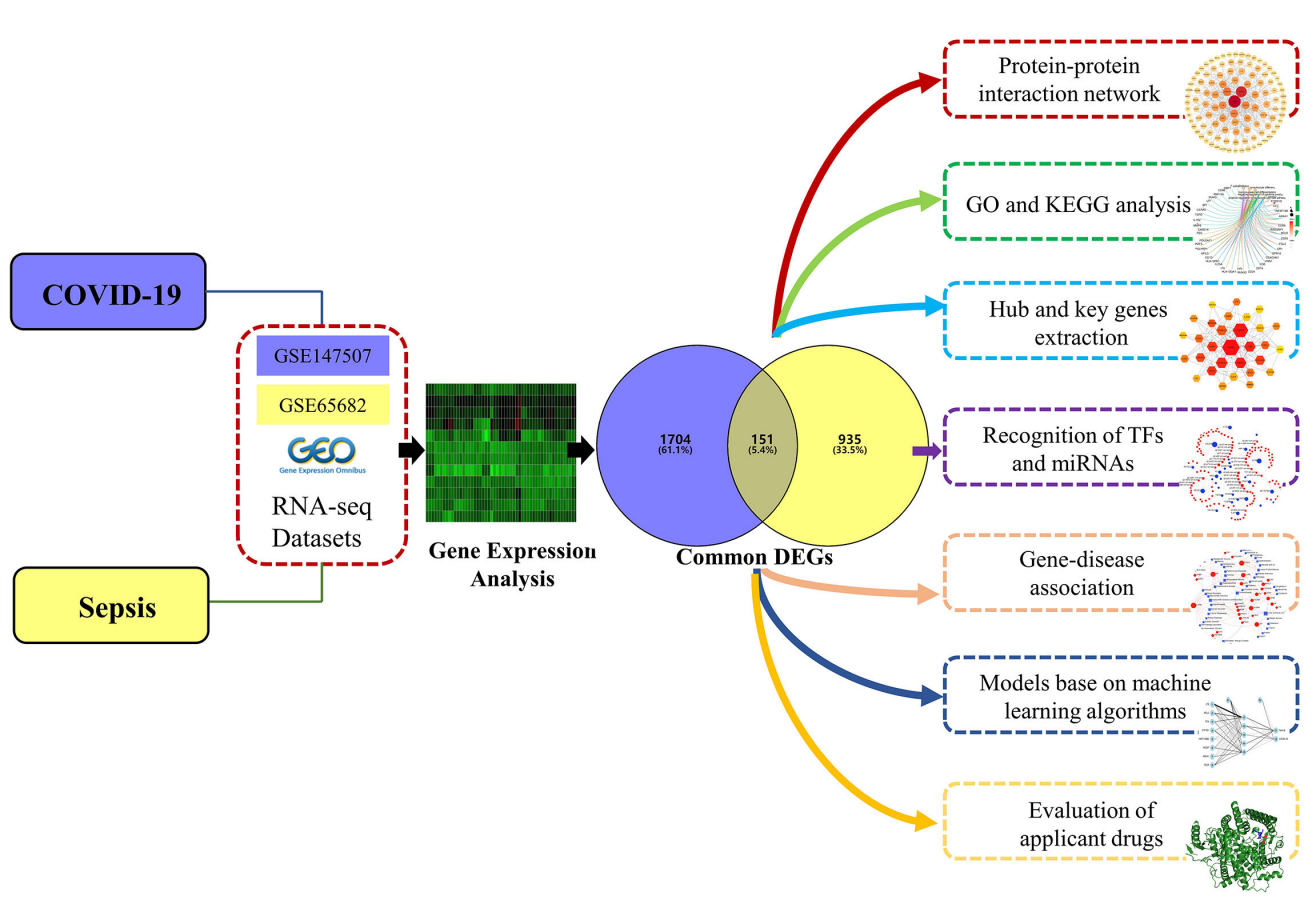
Schematic illustration of the overall general workflow of this study.

This study aims to understand the common pathogenesis between COVID-19 and sepsis, and to unearth potential drugs. First, datasets from the GEO database for COVID-19 and sepsis were analyzed to identify differentially expressed genes (DEGs) for these two diseases, and then further compared to obtain common DEGs. Based on the common DEGs, the enriched pathways and functions of these genes were analyzed to understand the biological processes they were involved in. Next, the protein–protein interaction (PPI) network was drawn to show the relationship between all DEGs, and the key genes with the highest degree of interaction were screened out from the Hub genes as potential biomolecules. The biological role of this key gene in COVID-19 was then analyzed to explore its potential mechanism in disease development and progression. In addition, a disease diagnosis model and risk prediction nomogram of COVID-19 were established using machine learning algorithms. Next, the transcriptional regulatory network of these common DEGs in COVID-19 was analyzed. Finally, we predict drugs related to common DEGs, providing new ideas for the treatment of COVID-19. The sequential workflow of our research is presented in **Figure 1**.

## Materials and methods

### Transcriptomic data acquisition

To determine shared genetic interrelations between COVID-19 and sepsis, three RNA-Sequencing datasets were downloaded from the Gene Expression Omnibus (GEO) database of the National Center for Biotechnology Information (NCBI) (https://www.ncbi.nlm.nih.gov/geo/) (17). The GEO accession ID of the COVID-19 dataset was GSE147507, which included transcriptional profiling from 78 samples (23 COVID-19 samples and 55 healthy control samples) through high throughput sequencing Illumina NextSeq 500 platform for extracting RNA sequence (18). The sepsis dataset having association number GSE65682 was based on GPL13667[HG-U219] Affymetrix Human Genome U219 Array platform, and contained 802 samples including healthy controls, non-sepsis critically ill patients and sepsis patients. Furthermore, the sepsis patients could be further categorized into pneumonia sepsis (n=192), abdominal sepsis (n=51) and others (n=443) based on infection site (19). According to some scholars, COVID-19 is a systemic infection, and its clinical manifestations range from asymptomatic to mild respiratory tract infection and influenza-like illness, to severe diseases with lung injury, multiple organ failure and death (20). However, the lung is thought to be the main site of SARS-CoV-2 infection and replication (14). Therefore, in our study, we screened 192 pneumonia sepsis samples and 42 healthy control samples from GSE65682 discovery dataset for further analysis. Besides, the GSE196822 discovery dataset was used as a validation cohort for development of the COVID-19 diagnostic model. This second selected COVID-19 dataset consisted of 40 samples from COVID-19 subjects and 9 healthy controls. which were sequenced using microarrays called Illumina HiSeq 4000 platform. The summarized information of the datasets was shown in **Table S1**.

### Differential gene expression analysis

Firstly, the DEGs for the corresponding diseases were extracted from two mRNA datasets (GSE147507 and GSE65682). Specifically, the DEGs were identified by using the “limma” R package and the Benjamini–Hochberg false discovery rate method was used to discover genes which were statistically significant and limit false positives (21). Genes exhibiting an adjusted P-values of <0.05 along with |log2FC|≥1.0 were identified as statistically significant genes. The mutual DEGs of GSE147507 and GSE65682 was acquired through an online VENN analysis tool called Jvenn (http://jvenn.toulouse.inra.fr/app/index.html) (22).

### Functional insights into the differentially expressed genes

To clarify potential biological mechanisms between COVID-19 and sepsis, we attempted to investigate the gene ontology (GO) terms and Kyoto Encyclopedia of Genes and Genomes (KEGG) enrichment pathways base on common DEGs. KEGG is considered as a knowledge base for systematic analysis of gene functions, linking genomic information with higher order functional information (23). Additionally, GO, a community-based bioinformatics resource, can provide information about gene product function by presenting biological knowledge as ontologies (24). GO analysis was classified into three subgroups, including molecular function (MF), biological process (BP) and cellular component (CC) (25). For quantifying the top listed functional items and pathways, a”clusterProfiler” R package was used to perform functional enrichment analysis, and a statistical threshold criterion with an adjusted P-value <0.05 was used to identify significant GO terms and KEGG pathways.

### Protein–protein interaction analysis and hub genes extraction

Proteins conclude their journey into a cell with a similar protein affiliation formed by a protein–protein network, which indicates the protein mechanisms (21). In this study, the protein subnetworks on common DEGs were identified to discover the associations between different diseases from the perspective of protein interactions. Specifically, an online analysis tool called STRING (https://www.string-db.org/) (version 11.5) was to insert common DEGs to generate PPI networks. Supported by Damian Szklarczyk, the STRING is a database which aims to integrate all known and predicted associations between proteins, including both physical interactions as well as functional associations (26). A combined score larger than 0.4 was used to construct the PPI network of frequent DEGs in this experiment. Then, the Cytoscape (version 3.9) was used for visual representation and further PPI network experimental studies. Furthermore, a Cytoscape plugin, CytoHubba (https://apps.cytoscape.org/apps/cytohubba), was put into practice to extract hub genes. Cytohubba is a significant Cytoscape application, which can rank and extract central or potential or targeted elements of a biological network based on various network features (21). Moreover, Cytohubba has 11 methods for investigating networks from various viewpoints, and Maximal Clique Centrality (MCC) is the best of them (27). The MCC function of Cytohubba was carried out to confirm the top 30 hub genes from the PPI network.

### Regulatory analysis of the key gene

Based on the analysis results of PPI network and Hub gene extraction, we further explored the biological function and possible mechanism of *ITGAM*, the most critical gene located at the core of the protein interaction network. First, the “limma” R package was used to implement differential expression analysis in GSE147507 discovery dataset to determine whether *ITGAM* differed between COVID-19 and healthy controls. Gene co-expression is a type of analysis method that uses a large amount of gene expression data to construct correlations among genes and thus discover the function of genes (28). Next, based on gene co-expression analysis, ITGAM-related gene regulatory networks were constructed using gene expression data from COVID-19 dataset. In addition, to further explore the potential pathways and molecular biological functions that *ITGAM* may affect in COVID-19, gene set enrichment analysis (GSEA) for *ITGAM* was performed in GSE147507 discovery dataset. GSEA includes pathway analysis and gene ontology analysis, which plays an essential role in extracting biological insight from genome-scale experiments (29, 30). Furthermore, immune correlation analysis of *ITGAM* was conducted. Specifically, based on ITGAM’s expression data, the COVID-19 samples were divided into high- and low-expressed groups using the mean of *ITGAM* expression levels as a zero cut-off. Next, the difference for immune cell infiltration between the high- and low-expressed groups was analyzed using the “CIBERSORT” R package, and the correlation between *ITGAM* and immune cells was further explored (31). Finally, differences in the expression of immune checkpoints between the high- and low- expressed groups and the correlation between *ITGAM* and immune checkpoints were analyzed by the “corrplot” R package.

### Developing diagnostic signature and risk model for COVID-19

Based on the common DEGs obtained by differential expression and VENN analysis, machine learning methods were used to screen the features/key genes and further constructed the diagnostic model and risk prediction model for COVID-19. Specifically, random forests (RFs) were applied to screen diagnostic features in GSE147507 discovery dataset. RF is one type of very popular ensemble learning method in which numerous randomized decision trees are constructed and combined to form an RF that is then used for classification or regression (32). In this study, the DEGs with Gini index > 1.0 were considered characteristic variables. Next, using GSE147507 discovery dataset as training cohort, artificial neural networks (ANNs) were performed to construct COVID-19 diagnostic model based on the signature genes. ANNs are a set of technologies often encompassed with artificial intelligence that attempt to simulate the function of the human brain, and have been applied in almost every aspect of medicine (33, 34). Further, the GSE196822 discovery dataset was used as a validation cohort to evaluate the performance of the diagnostic model. Finally, a nomogram was developed based on the results of RF analysis to calculate the risk of COVID-19 for an individual patient by the points associated with the risk factors, and the performance of the nomogram was assessed by decision and calibration curve.

### Identification of transcription factors and miRNAs

To determine the major variation at the transcriptional level and gain a deeper understanding of the key protein regulatory molecules or common DEG, the DEG–miRNA (microRNA) interaction networks and transcription factor (TF)–DEG interaction networks were identified in our analysis. Specifically, the NetworkAnalyst platform was utilized to locate topologically credible TFs from the JASPAR database that tend to bind to the common DEGs (21). For DEG–miRNA network construction *via* NetworkAnalyst platform, the TarBase (35) and miRTarBase (36) databases were used to extracted miRNAs with common DEGs focused on topological analysis (37).

### Gene–disease association analysis

DisGeNET is a knowledge management platform, which integrates and standardizes the data about disease associated genes and variants from multiple sources, including the scientific literature (38).The gene-disease relationship network was established through NetworkAnalyst platform to uncover associated diseases and their chronic complications related to the common DEGs (21).

### Evaluation of applicant drugs

In this analysis, the protein–drug interaction (PDI) and identified pharmacological molecules were predicted by using the common DEGs that COVID-19 shares with sepsis. The web portal od Enrichr and the Drug Signatures Database (DSigDB) were used to analyze the drug moleculars based on the DEGs from both COVID-19 and sepsis. Enrichr (http://amp.pharm.mssm.edu/Enrichr) contains a large collection of diverse gene set libraries available for analysis and download, which can be used to explore gene-set enrichment across a genome-wide scale (39). DSigDB is a new gene set resource for gene set enrichment analysis, which related drugs/compounds and their target genes. The DSigDB database was accessed through Enrichr under the Diseases/Drugs function (40).

### Molecular docking simulation

Molecular docking that an established in silico structure-based method is widely used in drug discovery. Docking enables the identification of novel compounds of therapeutic interest, predicting ligand-target interactions at a molecular level, or delineating structure-activity relationships (SAR), without knowing *a priori* the chemical structure of other target modulators (41). In our study, key targets of COVID-19 were obtained through literature search, including ACE2, 3CLpro, M^pro^, PLpro and RdRp. Next, the crystal structures of these key proteins were downloaded from the Protein Data Bank (https://www.rcsb.org/) for further molecular docking. The PDB codes for these five key proteins are shown below: *1R42* for ACE2, *6LU7* for 3CLpro, *5B60* for Mpro, *6Y2E* for PLpro, *6NUS* for RdRp. In addition, the molecular structures of potential drug molecules were obtained from the ZINC (https://zinc.docking.org/) database. The Autodock tools (version 1.5.4) was utilized in all the docking experiments, with the optimized model as the docking target. The screening method is restricted to molecular docking, and molecular dynamics simulation has not been carried. In addition, the results were shown with binding energy (BE), a weighted average of docking score, to assess the reliability and describe the accuracy of the ligand positioning. Pymol (PyMOL Molecular Visualization System 2020) was used for 3D visualization of the docking results.

## Results

### Identification of common transcriptional signatures between COVID-19 and sepsis

**Figure 2:**
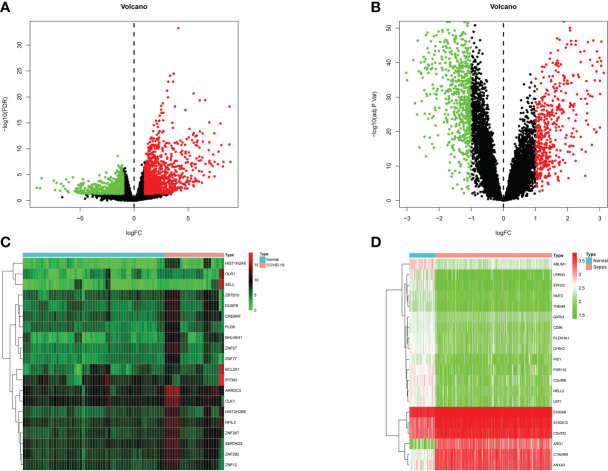
Volcano plots exhibit differentially expressed genes (DEGs) of **(A)** COVID-19 and **(B)** sepsis. Red dots indicated up-regulated genes, blue dots indicated down-regulated genes, and gray dots indicated non-DEGs, with FC≥1.0 and P-value<0.05. Heatmaps show the result of clustering analysis based on DEGs for **(C)** COVID-19 and **(D)** sepsis.

Patients with severe COVID-19 may develop a systemic inflammatory response syndrome (SIRS) that may progress to sepsis if inflammation worsens. To examine the interrelationships and implications between COVID-19 and sepsis, the human RNA-seq dataset and microarray datasets were analyzed from the GEO to identified the disrupting genes that trigger COVID-19 and sepsis. a total of 1855 DEGs were obtained from the COVID-19 dataset, including 1206 up-regulated DEGs and 649 down-regulated genes. In addition, a total of 1086 DEGs were identified in the sepsis blood dataset by differential expression analysis, of which 481 genes were up-regulated and 605 genes were down-regulated (**Table S1**). The two volcano plots in **Figure 1** visually demonstrated the overall picture of transcribed gene expression for COVID-19 and sepsis, where red and blue dots indicated up- and down-regulated genes with significant differences, respectively **(** **Figures 2A, B** **)**. Furthermore, we employed heatmaps to present the results of cluster analysis and expression analysis of the top 20 DEGs among different samples in COVID-19 and sepsis datasets, respectively (**Figures 2C, D**). The top 20 DEGs for COVID-19 included *HIST1H2AK*, *OLR1*, *SELL*, *ZBTB10*, *DUSP8*, *CREBRF*, *PLD6*, *BHLHE41*, *ZNF57*, *ZNF77*, *BCL2A1*, *IFITM2*, *ARRDC3*, *CLK1*, *HIST2H2BE*, *NFIL3*, *ZNF267*, *SERTAD2*, *ZNF292* and *ZNF12*. In sepsis discovery set, the top 20 DEGs were *ABLIM1*, *LRRN3*, *EPHX2*, *NMT2*, *THEM4*, *GATA3*, *CD96*, *PLEKHA1*, *DYRK2*, *PID1*, *P2RY10*, *C2orf89*, *NELL2*, *LEF1*, *S100A8*, *S100A12*, *C5orf32*, *ARG1*, *C19orf59* and *ANXA3*.The identification of these genes with significant differential expression could help us to obtain a critical entry point for studying the development of diseases, which in turn could help to understand the underlying mechanisms of diseases and to obtain new therapeutic targets. After performing the cross-comparative analysis on the Jvenn, a reliable web portal for Venn analysis, a total of 151 common DEGs were identified from COVID -19 and sepsis datasets (**Figure 4A**). The results of differential expression analysis suggested that there were some mechanistic commonalities and interaction between COVID-19 and sepsis.

### Pathway enrichment and gene ontology analysis

**Figure 3:**
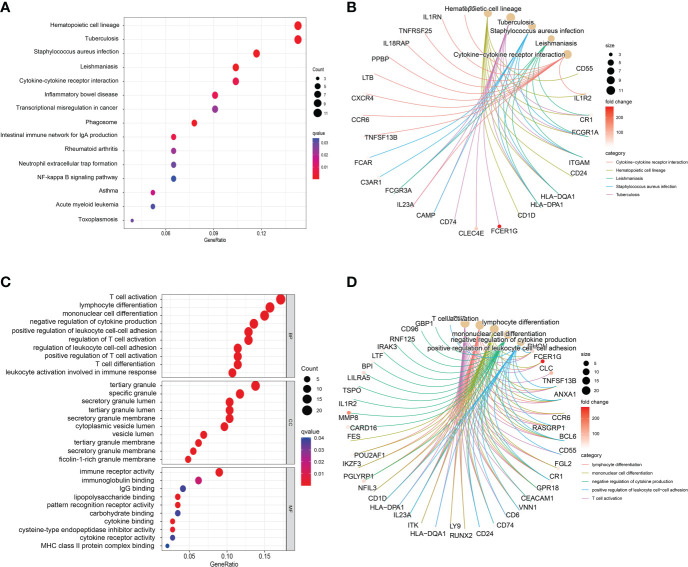
Bubble graphs indicate the results for **(A)** Kyoto Encyclopedia of Genes and Genomes (KEGG) and **(C)** Gene Ontology (GO) analysis based on the common differentially expressed genes (DEGs). Loop graphs show the correlation between the five most important **(B)** pathways or **(D)** GO terms and the enriched DEGs.

To further understand the biological functions and signaling pathways involved in these common DEGs, we implemented KEGG pathway enrichment and GO functional analysis. The top 15 important pathways were displayed with bubble plots (**Figure 3A**). From the results of KEGG pathway analysis, these 151 common DEGs were mainly enriched in infectious/inflammatory disease-related and immune response-related pathways, for example, Staphylococcus aureus infection, Inflammatory bowel disease, Cytokine-cytokine receptor interaction pathway and NF-kappa B signaling pathway. It is well-known that both COVID-19 and sepsis are associated with inflammatory and immune responses in the body, which play an important role in the development and progression of these two diseases, and are closely related to the therapeutic effect and prognosis of patients (3, 14, 42). Our pathway analysis results also showed that the immune-related pathway, Cytokine-cytokine receptor interaction, was the most significantly enriched pathway (**Figure 3B**), suggesting that these common DEGs may affect the progression of the disease through immune-related biological functions or signaling pathways.

GO analysis is divided into three parts: MF, BP and CC. **Figure 3C** presented the top 10 GO terms for MF, BP, and CC, respectively. Specific analysis revealed that the top 10 GO terms of BP were all associated with immune function, such as T cell activation, lymphocyte differentiation, mononuclear cell differentiation and negative regulation of cytokine production. Interestingly, most of the BP terms were associated with T cell immune function. In addition, the results of CC showed that these common DEGs were mainly involved in the formation or release of intracellular granules, for example, tertiary granule, specific granule, specific granule lumen and cytoplasmic lumen vesicle. Previous studies have suggested that tertiary granule and specific granule are associated with the function of human mature neutrophils, including differentiation and pro-inflammatory effect of neutrophils (43). During inflammation, neutrophils are activated and secrete part of the granular contents, which are cytotoxic and in part responsible for the collateral damage associated with neutrophil tissue infiltration (44). Furthermore, the results of MF analysis presented that MF terms were also mainly associated with immune responses, including immune receptor activity, immunoglobulin binding and IgG binding. **Figure 3D** shows the correlation between the five most important GO terms and the enriched DEGs, including lymphocyte differentiation negative regulation of cytokine positive production regulation of leukocyte cell−cell adhesion T cell activation, all of which are immune-related molecular functions. Similar to the results suggested by KEGG analysis, these common DEGs may involve immune-related functions and pathways of the body, which in turn affect the disease progression of COVID-19.

### Protein–protein interaction network analysis and identification of hub genes

**Figure 4:**
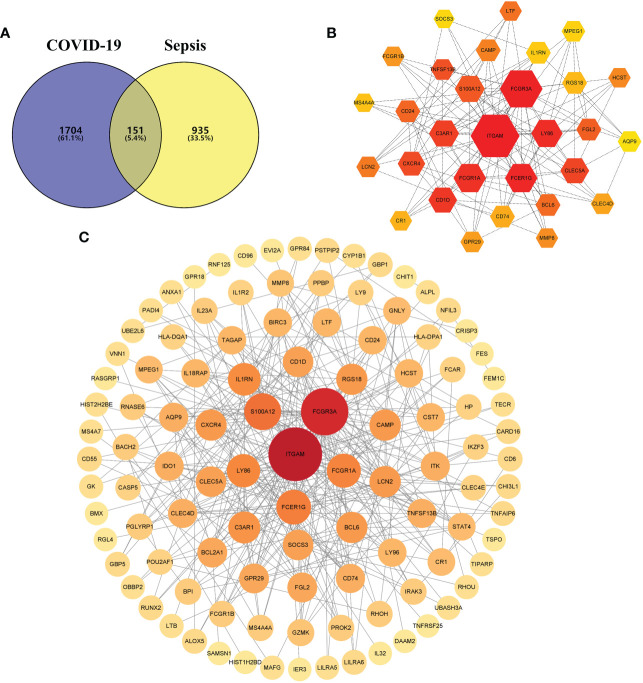
**(A)** The Venn diagram depicts the shared differentially expressed genes (DEGs) between COVID-19 and sepsis. **(B)** The top 30 hub gene was identified from the protein–protein interaction (PPI) network, and the hexagonal nodes represent DEGs and edges represent the interactions between nodes. **(C)** The (PPI) network of common DEGs among COVID-19 and RA, and the circle nodes represent DEGs and edges represent the interactions between nodes.

A PPI network was constructed using the common DEGs among COVID-19 and sepsis. The PPI network visually demonstrates the intercorrelations between different proteins, suggesting the underlying mechanisms by which proteins function. The assessment and analysis of PPI networks can help to obtain key proteins that influence the biological functions of cells and systems (45). Based on the online analysis website, STRING, the PPI network of proteins derived from shared DEGs was constructed to portray functional and physical interactions between COVID-19 and sepsis. The PPI network of common DEGs included 151 nodes and 322 edges and was depicted in **Figure 4C**, with the PPI enrichment p-value < 0.001. As shown in the figure, the size and color depth of the circles indicated the degree of intercorrelation of the proteins, and the more connections to the central proteins, the stronger the relationship, suggesting its importance. By using cytoHubba package of Cytoscape, the top 30 (19.87%) DEGs were considered as the most influential genes. The top 30 influential genes included *ITGAM, FCGR3A, S100A12, FCER1G, FCGR1A, LY86, IL1RN, C3AR1, LCN2, BCL6, CAMP, RGS18, CXCR4, CLEC5A, SOCS3, CD1D, FGL2, GPR29, AQP9, CLEC4D, CD74, TNFSF13B, CD24, LTF, HCST, MPEG, CR1, MMP8, MS4A4A* and *FCGR1B*, with specific information showing in **Table S2**. The identification of hub genes from common DEGs facilitates us to obtain more critical signatures in order to discover potential biomarkers. Since hub genes were potential, a submodule network was constructed by the Cytohubba plugin’s aid to deeper understand their near connectivity and proximity (**Figure 4B**).

**Figure 5:**
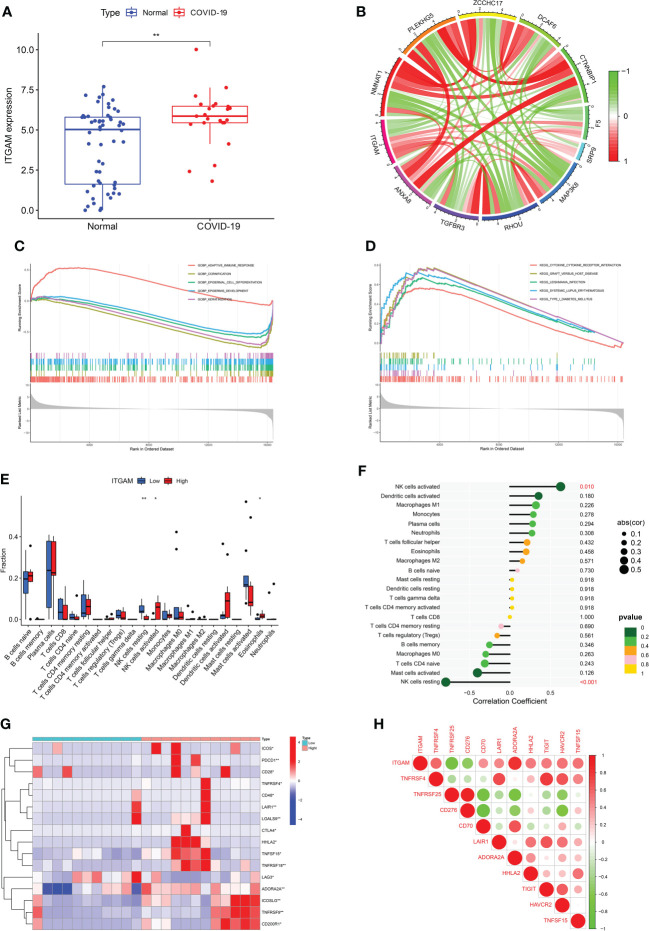
**(A)** The differential expression levels of *ITGAM* between COVID-19 and healthy controls. **(B)** The construction of a co-expression network for *ITGAM* with other genes in COVID-19. Gene set enrichment analysis (GSEA) for ITGAM in COVID-19, including **(C)** gene ontology analysis and **(D)** pathway analysis. **(E)** The difference for immune cell infiltration between ITGAM high- and low-expressed groups in COVID-19. *p < 0.05; **p < 0.01. **(F)** Plots of immune cells associated with ITGAM in COVID-19. **(G)** The clustering analysis for differentially expressed immune checkpoints between ITGAM high- and low-expressed groups in COVID-19. **(H)** Heatmap of immune checkpoints associated with ITGAM in COVID-19.

From the sub-module network of hub genes, *ITGAM* was shown to have the most edges, that is, the most proteins associated with it, so in further studies, the biological role of *ITGAM* in COVID-19 was focused to explore its potential mechanism in the development and progression of the disease. *ITGAM* encodes integrin-αM (CD11b +), molecule that combines with integrin-β2 to form a leucocyte-specific integrin, which associated with multiple immune disorders (46). The expression difference analysis indicated that the expression level of *ITGAM* was significantly different between COVID-19 and normal samples, with p-value < 0.01 (**Figure 5A**), suggesting that this signature may have an important role in COVID-19. In addition, a co-expression network of *ITGAM* with other genes was constructed by Gene Co-expression Network Analysi**s** (GCNA) **(** **Figure 5B**). The GCNA results showed that *ITGAM* had a significant correlation with 156 genes (p-value < 0.05), and **Figure 4B** showed only the top 11 important genes, including *TGFBR3*, *NMNAT1*, *RHOU*, *PLEKHG5*, *MAP3K8*, *ZCCHC17*, *DCAF6*, *CTNNBIP1*, *SRP9*, *F5*, and *S100A2*. Among them, *TGFBR3*, *RHOU*, *MAP3K8*, *SRP9* and *F5* were positively correlated with *ITGAM* expression, and the remaining six genes were negatively correlated with *ITGAM*. As a result of GCNA, the co-expressed genes mediated the expression and function of *ITGAM* through different pathways, and *ITGAM* played multiple biological roles *in vivo*. Furthermore, GASE enrichment analysis was implemented to interrogate the function and pathway of *ITGAM*. Among the GO terms, the GSEA analysis in the GSE147507 dataset revealed that the samples of highly expressed *ITGAM* were mainly enriched in an important immune-related biological process, adaptive immune response. Other GO terms involved included cornification, epidermal cell differentiation, epidermis development and keratinization (**Figure 5C**). Among the KEGG pathways, the top 5 signaling pathways influenced by highly expressed *ITGAM* were cytokine-cytokine receptor interaction, graft versus host disease, leishmania infection, systemic lupus erythematosus and type I diabetes mellitus (**Figure 5D**). In addition, based on the average expression level of *ITGAM*, the COVID-19 samples from the GSE14750 dataset were divided into high- expressed and low- expressed groups. In order to explore the degree of immune cell infiltration between the high and low *ITGAM* expression groups to understand the potential immune mechanism, the infiltration levels of 22 immune cells were analyzed between the two groups using the “CIBERSORT” R package. The results of “CIBERSORT” analysis showed that there were significant differences in resting NK cells, activated NK cells and Eosinophils between the *ITGAM* high-expressed and the low-expressed groups (**Figure 5E**). Interestingly, our results showed that *ITGAM* was positively correlated with activated NK cells, but negatively correlated with resting NK cells, with a p-value < 0.01 (**Figure 5F**). Finally, the expression levels of immune checkpoints were analyzed between the *ITGAM* high-expressed and the low-expressed groups and found that a total of 16 immune checkpoint genes were differentially expressed, including *ICOS*, *PDCD1*, *CD28*, *TNFRSF4*, *CD48*, *LAIR1*, *LGALS9*, *CTLA4*, *HHLA2*, *TNFSF15*, *TNFRSF18*, *LAG3*, *ADORA2A*, *ICOSLG*, *TNFRSF9* and *CD200R1* (**Figure 5G**). Among them, the study results suggested that 14 immune checkpoints (*TNFRSF4*, *CD48*, *TNFRSF18*, *CD70*, *HHLA2*, *BTLA*, I*COSLG*, *TNFRSF9*, *CTLA4*, *LGALS9*, *PDCD1*, *TNFSF15*, *LAIR1*, A*DORA2A*) were positively correlated with *ITGAM*, while *CD276* and *TNFRSF25* were negatively correlated with *ITGAM* (**Figure 5H**). The results of the above analysis help us to preliminarily understand the immune mechanism of *ITGAM* in COVID-19, which in turn taps its potential biological functions.

### Construction of disease diagnosis and risk model based on common DEGs

**Figure 6:**
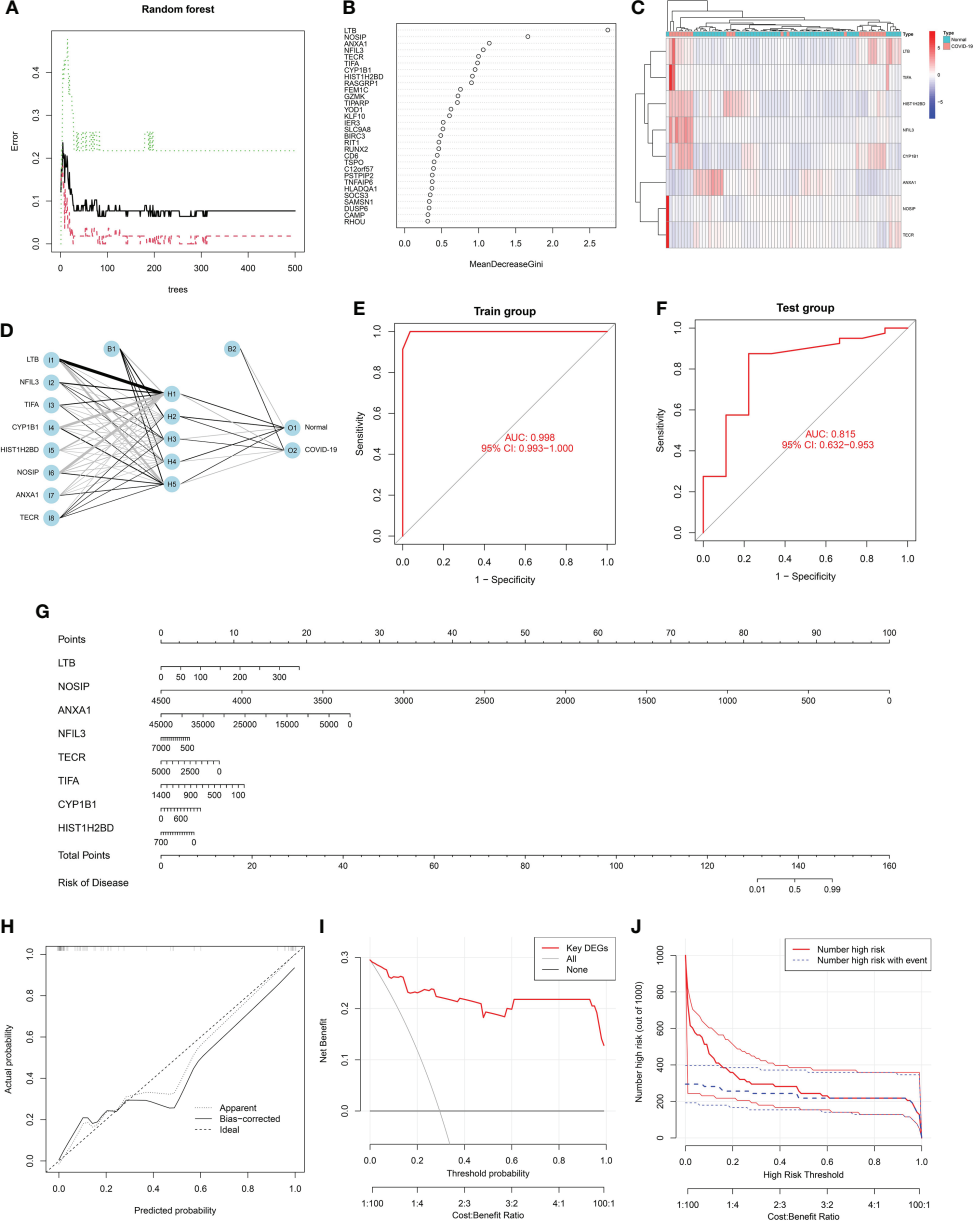
Screening feature genes from common differentially expressed genes (DEGs) using random forest (RF): **(A)** The random forest trees; **(B)** The importance rankings of features. **(C)** Heatmap shows the clustering analysis results for feature genes in COVID-19. Red represented up-regulated genes and blue represented down-regulated genes. **(D)** Graph represents the disease diagnosis model constructed by artificial neural network (ANN). Receiver operating characteristic (ROC) curve analysis of the model’s performance for **(E)** training set and **(F)** validation set, respectively. **(G)** A constructed nomogram for risk prediction of COVID-19. **(H)** The calibration curve, **(I)** decision curve analysis (DCA) curve and **(J)** clinical impact curve for assessing the nomogram’ performance.

Through the above analysis, it was found that the common DEGs of COVID-19 and sepsis may affect the disease process of COVID-19 through different functions and pathways, therefore, based on 151 common DEGs, we screened and constructed a diagnostic model and risk model of COVID-19 using machine learning algorithms. Specifically, the RF analysis was implemented to select key DEGs, and selected the top eight important DEGs for model construction according to the variable importance ranking, with a Gini index > 1.0 (**Figures 6A, B**). **Figure 6C** visually showed the expression levels of these eight key genes in COVID-19 and normal samples. Next, based on eight key signatures, a disease diagnostic model for COVID-19 was constructed in the training set, with an AUC = 0.998 (**Figures 6D, E**). In addition, the GSE196822 dataset was used as a validation cohort to further assess the performance of this ANN model and found that it performed well in the validation set (**Figure 6F**). The above diagnostic model has a good discriminatory ability for COVID-19 and hopes to be applied in clinical practice to assist the clinical diagnosis of COVID-19. Furthermore, a nomogram for COVID-19 disease risk assessment was successfully established by using the above eight key signatures for easier use (**Figure 6G**). Then, the accuracy of this nomogram was preliminarily assessed using the calibration curve, and the results showed that the Bias-corrected curve coincided well with the Ideal curve (**Figure 6H**). Furthermore, both the DCA curve and clinical impact curve (**Figures 6I, J**) indicated that the risk model had good performance ability. Specifically, it can be seen from the above figure that the model can achieve a higher net benefit rate at a threshold around 0.6.

### Construction of regulatory networks at transcriptional level

**Figure 7:**
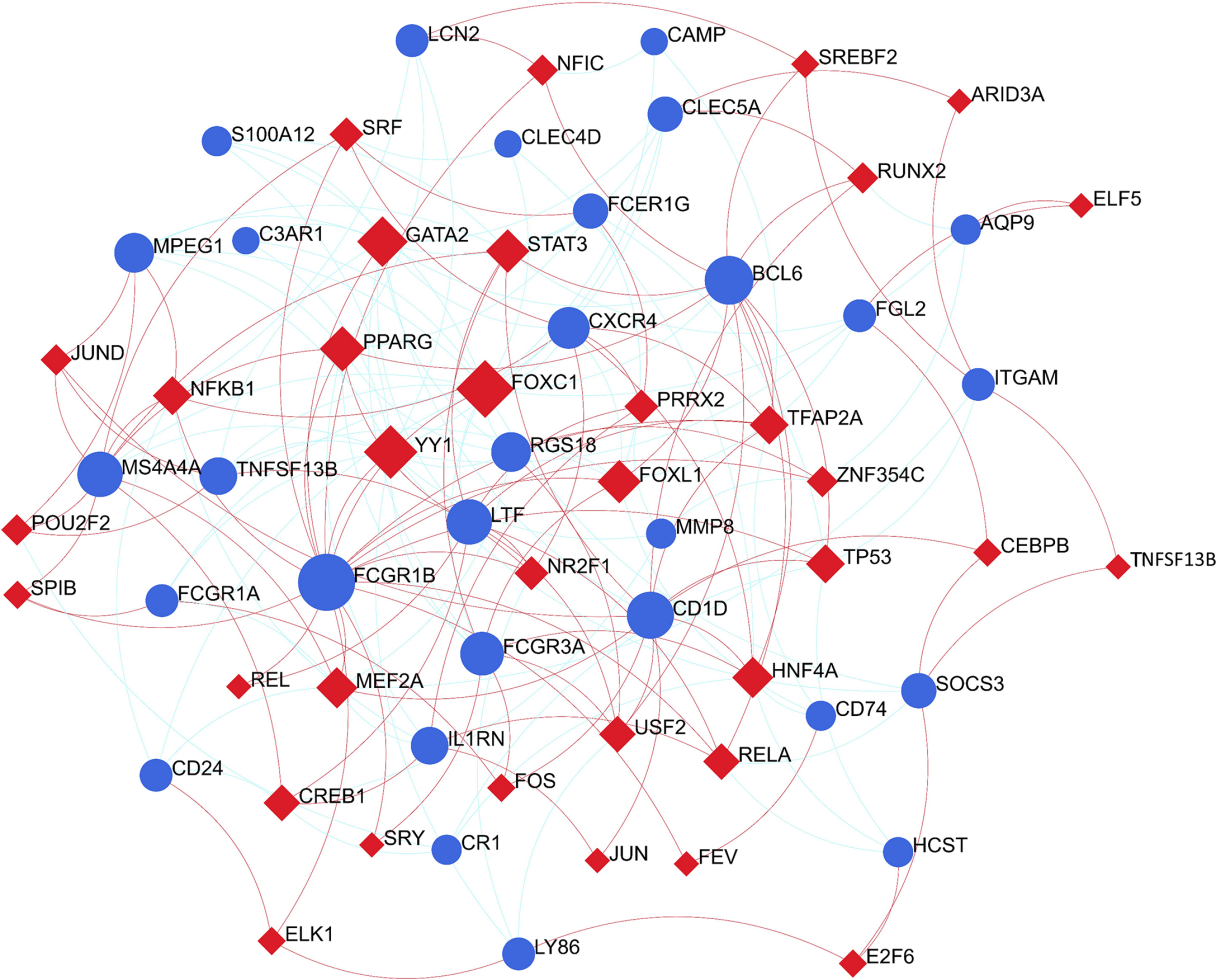
The construction of an interconnected regulatory interaction network for DEG-TFs. In this figure, circles represent common differentially expressed genes (DEGs), while diamonds are transcription factors (TFs).

**Figure 8:**
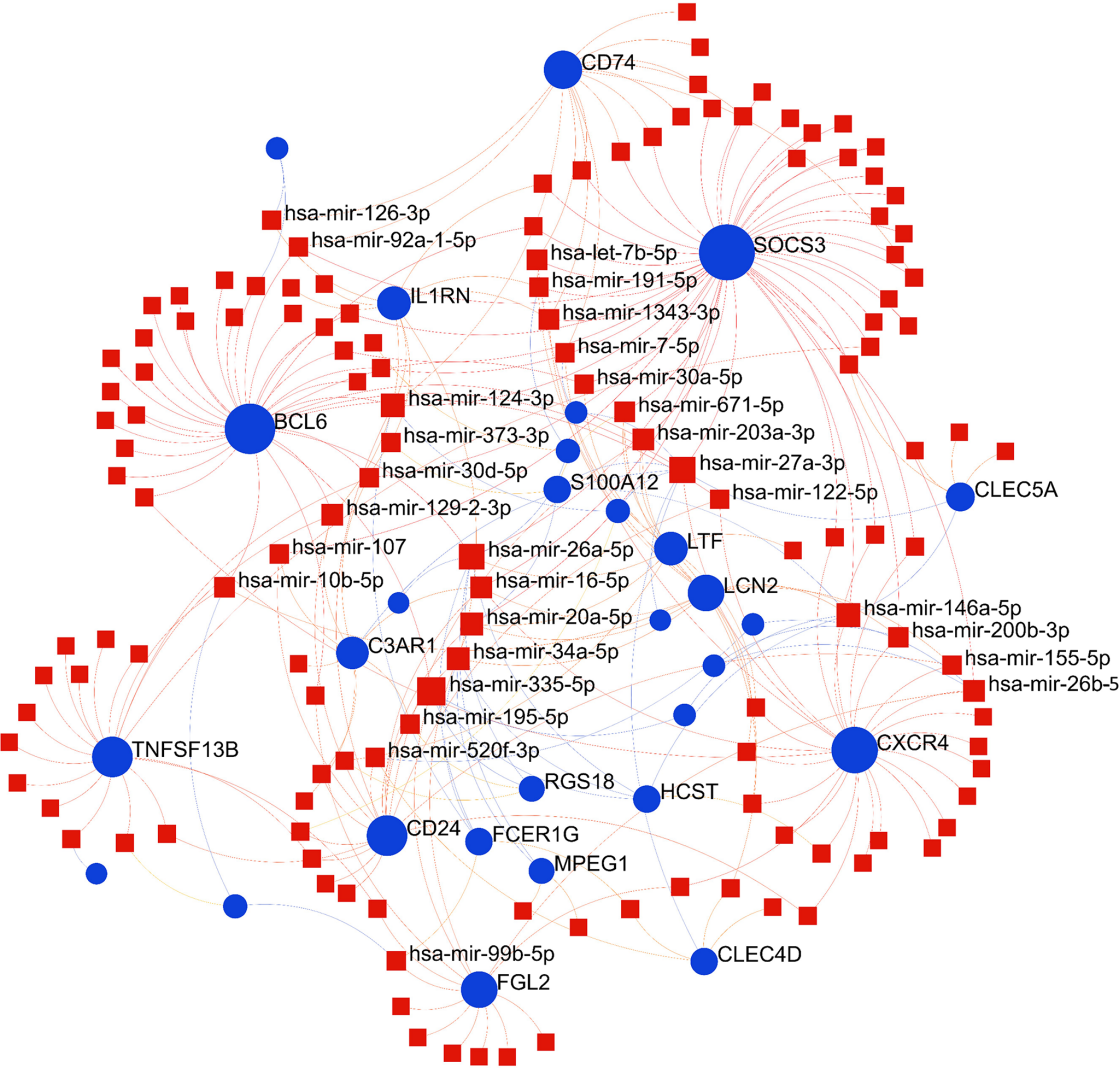
The construction of an interconnected regulatory interaction network for DEGs-miRNAs. In this figure, square nodes indicate miRNAs and circle nodes represent common differentially expressed genes (DEGs).

To identify substantial changes happening at the transcriptional level and get insights into the common DEGs, a network-based approach was employed to decode the regulatory TFs and miRNAs. The DEG–TFs interactions network was identified by using TarBase and miRTarBase bases and displayed in **Figure 7**. Circles represented common DEGs, while diamonds were TFs. The size of the circular or rhombus node depends on the degree of the node. The degree of a node is the number of connections the node has with other nodes in the network. Nodes with a higher degree are considered as important hubs of the network. From the **Figure 7**, *FCGR1B*, *BCL6*, *CD1D*, *MS4A4A* and *LTF* were more among more highly expressed DEGs as these genes have a higher degree in the TF–gene interactions network. TFs such as FOXC1, YY1, GATA2, PPARG and FOXL1 were more significant than others as presented in the same figure. Again, the **Figure 8** represented the interactions of miRNAs regulators with common DEGs. In the **Figure 8**, red squares represented miRNA s, while blue circles represented DEGs. Our results showed that *SOCS3*, *BCL6*, *CXCR4*, and *TNFSF13B* were the hub genes of this network, with the five genes most involved in miRNAs. Besides, the significant hub miRNAs were detected from the miRNAs-gene interaction network, namely hsa-mir-27a-3p, hsa-mir-26a-5p, hsa-mir-124-3p, hsa-mir-146a-5p and hsa-mir-20a-5p.

### Identification of disease association

**Figure 9:**
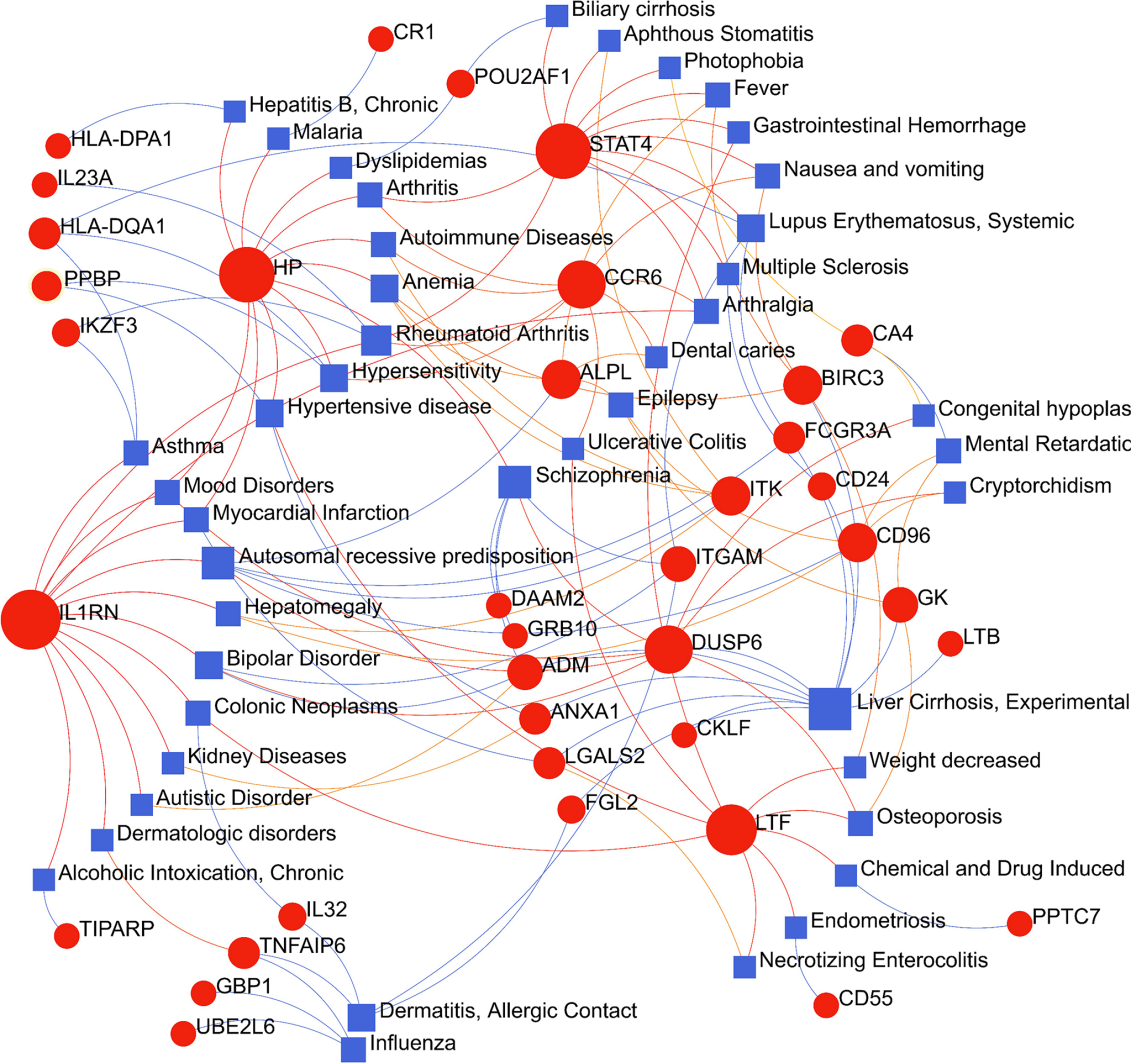
The gene-disease association network represents diseases associated with common differentially expressed genes (DEGs). The disorder is depicted by the square node and also its subsequent DEGs are defined by the circle node.

The circumstances in which different diseases can be correlated or associated are that they must usually have one or more similar genes (21). Therapeutic design strategies for disorders begin with deciphering the relationship between genes and disease (40). From the analysis of the gene-disease association base on the DisGeNET platform, it was noticed that liver Cirrhosis, rheumatoid arthritis, hypertensive disease, allergic contact dermatitis, lupus erythematosus systemic, anemia, hypersensitivity and Influenza were most coordinated to our reported hub genes, and even in COVID-19 (**Figure 9**). Interestingly, the study results suggested that most of the diseases mentioned above were related to inflammation or immune response in the body. The gene-disease association suggests that certain diseases may have the same molecular mechanism in progression, which has implications for our development of new therapeutic strategies for COVID-19.

### Identification of candidate drugs and target–chemical interaction in COVID-19

A chemical–protein interaction network is an important research tool for understanding the function of proteins, which is helpful for advancing drug discovery (29). In the aspects of common DEGs as potential drug targets in COVID-19 and sepsis, the candidate drugs were identified by using Enrichr based on transcriptome signatures from the DSigDB database. The top 10 drug molecules selected based on p-value were considered as potential compounds that could be used for COVID-19 treatment and subsequent analysis. These 10 possible drug molecules included cephaeline, mebendazole, tretinoin, progesterone, emetine, digitoxigenin, trichostatin A, piperlongumine, terfenadine and strophanthidin (**Table 1**). These potential drugs were recommended for use in the common DEGs, which was a common compound for the treatment of two diseases.

**Table 1:** Candidate drugs (top ten) identified from gene–drug interaction enrichment analysis.

Name	Adjusted P-value	Chemical Formula
**cephaeline** **mebendazole** **tretinoin** **progesterone** **emetine** **digitoxigenin** **trichostatin A** **piperlongumine** **terfenadine** **strophanthidin**	4.94E-10 9.95E-10 1.64E-09 8.06E-09 8.28E-09 2.71E-06 2.71E-06 3.62E-06 7.43E-06 1.67E-05	C_28_H_38_N_2_O_4_ C_16_H_13_N_3_O_3_ C_20_H_28_O_2_ C_21_H_30_O_2_ C_29_H_40_N_2_O_4_ C_23_H_34_O_4_ C_17_H_22_N_2_O_3_ C_17_H_19_NO_5_ C_32_H_41_NO_2_ C_23_H_32_O_6_

**Figure 10:**
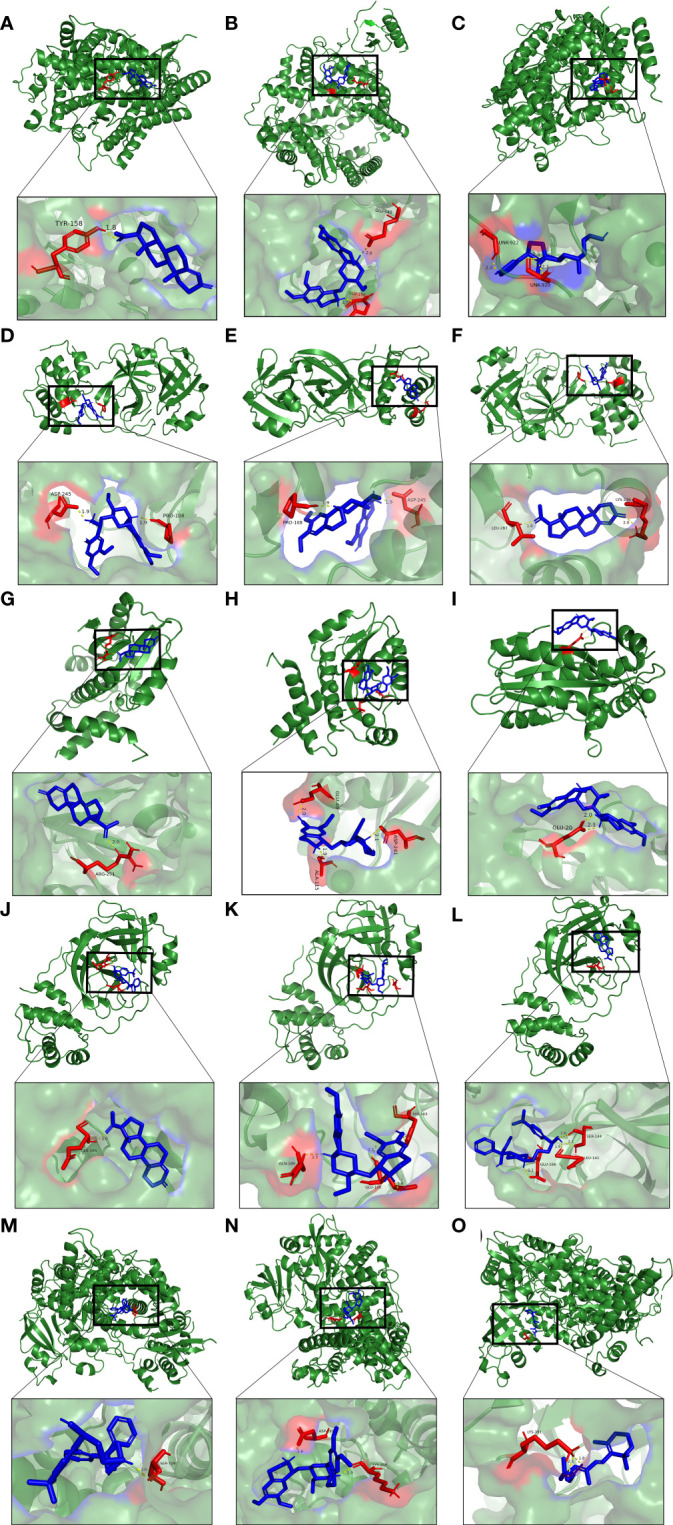
Molecular docking patterns for **(A)** progesterone, **(B)** emetine, **(C)** digitoxigenin with the ACE2, respectively. Molecular docking patterns for **(D)** emetine, **(E)** progesterone, **(F)** cephaeline with the 3CLpro, respectively. Molecular docking patterns for **(G)** progesterone, **(H)** cephaeline, **(I)** emetine with the M^pro^, respectively. Molecular docking patterns for **(J)** progesterone, **(K)** cephaeline, **(L)** terfenadine with the PLpro, respectively. Molecular docking patterns for **(M)** progesterone, **(N)** emetine, **(O)** tretinoin with the RdRp respectively.

Furthermore, molecular docking was implemented to predict the binding mode of these 10 potential compounds with five different targets from COVID-19, including ACE2, 3CLpro, M^pro^, PLpro and RdRp. It is generally believed that the lower the stabilization energy of ligand binding to the receptor, the greater the possibility of action, and the binding energy in screening criteria was changed to ≤−5.0 kcal/mol (-20 kJ/mol) in this study. The results of molecular docking analysis were shown in **Table 2**, and the binding energy of most compounds met the criteria. **Figure 10** demonstrated the binding differences of the top 3 potential compounds that bind to these 5 COVID-19 targets. The bioactive compounds, progesterone, emetine, and digitoxigenin, were the most promising compounds on ACE2, and emetine, progesterone and cephaeline were the most active on 3CLpro. For the main protease (M^pro^), the most promising compounds included progesterone, cephaeline and emetine. Besides, the most potential compounds binding to PLpro were progesterone, cephaeline and terfenadine, while progesterone, emetine and tretinoin were the most active on RdRp. Interestingly, emetine was found to have lower stabilization energy at binding sites to four targets (ACE2, 3CLpro, M^pro^, and RdRp), while progesterone could stably bind to all COVID-19 targets, with all binding energy ≤−6.5 kcal/mol. Therefore, these two drugs may be the most potential compounds for the treatment of COVID-19, and further studies on the pharmacological effects of these two compounds are needed.

**Table 2:** The binding sites and energies for key drug targets of COVID-19 were evaluated through AutoDock calculations.

Drug targets	Amino acid	Binding energy
**ACE2**		
cephaeline	ALA-348, ASP-350	-7.40
mebendazole	TYR-158, SER-254	-4.52
tretinoin	UNK-914, UNK-915, UNK-916, UNK-917	-5.90
progesterone	TYR-158	-8.08
emetine	GLU-140, GLU-150	-7.97
digitoxigenin	UNK-920, UNK-922	-7.48
trichostatin A	ASP-350, ARG-393, LYS-562	-4.51
piperlongumine	ASN-210	-5.28
terfenadine	GLU-564	-6.43
strophanthidin	SER-170, UNK-951	-5.13
**3CLpro**		
cephaeline	PRO-108, ASP-245	-6.59
mebendazole	GLU-166, PRO-168	-3.80
tretinoin	LYS-97	-6.01
progesterone	LYS-236, LEU-287	-7.00
emetine	PRO-108, ASP-245	-7.02
digitoxigenin	LYS-137, LEU-272, LEU-287	-6.55
trichostatin A	LYS-97, ASN-119, GLY-120	-3.79
piperlongumine	GLU-166, PRO-168	-5.45
terfenadine	ASP-33	-5.41
strophanthidin	LYS-137, LEU-287	-5.06
**M^pro^**		
cephaeline	ALA-115, GLU-49, ASP-241	-7.15
mebendazole	ALA-189, ASP-191	-4.86
tretinoin	SER-253	-6.60
progesterone	ARG-251	-7.52
emetine	GLU-20	-7.05
digitoxigenin	ALA-115	-6.17
trichostatin A	GLY-215, GLN-224	-3.38
piperlongumine	ASP-241	-4.90
terfenadine	GLU-20	-5.87
strophanthidin	GLY-93	-5.58
**PLpro**		
cephaeline	HIS-163, GLU-166, GLN-189	-7.22
mebendazole	TYR-239, MET-276, GLY-278, ALA-285	-4.94
tretinoin	LYS-97	-5.87
progesterone	GLU-166	-7.57
emetine	GLY-120	-6.45
digitoxigenin	ASN-142, GLU-166	-6.96
trichostatin A	ARG-298, GLN-299	-4.24
piperlongumine	THR-26, GLY-143	-4.93
terfenadine	GLU-166, LEU-141, SER-144	-7.03
strophanthidin	GLU-240, HIS-246	-5.71
**RdRp**		
cephaeline	ASP-284, ASP-291	-6.06
mebendazole	THR-141	-3.69
tretinoin	LYS-391	-6.56
progesterone	SER-709	-7.36
emetine	LYS-288, ASP-291	-7.20
digitoxigenin	ILE-266, THR-319	-6.42
trichostatin A	ASP-336	-3.05
piperlongumine	LYS-603	-3.56
terfenadine	SER-709	-4.24
strophanthidin	ASP-284, ASP-291, GLN-292, TYR-294	-3.05

## Discussion

From the COVID-19 pandemic to the present, research on COVID-19 has become more and more in-depth, and it has been found that COVID-19 has many unique characteristics, and many manifestations are very similar to sepsis (47). For example, both cytokines and chemokines are elevated in the serum of severe COVID-19 patients, and similar manifestations are seen in sepsis patients. Severely ill COVID-19 patients have clinical manifestations of shock without hypotension. At the same time, a hypercoagulable state is present in both diseases. At present, many pathological studies believe that sepsis is caused by the imbalance between the body’s pro-inflammatory response and anti-inflammatory response (48). According to some scholars, treating COVID-19 as viral sepsis, using effective antiviral therapy for patients, regulating innate and adaptive immune responses, and limiting their damage to tissues will help improve the treatment outcome (7). The focus of this study is to explore the correlation between COVID-19 and sepsis, and to explore the common mechanisms that may be involved between the two, so as to provide a theoretical basis for the classification and treatment of COVID-19.

The enrichment analysis of pathways and functions helps us to understand the regulatory effects and specific mechanisms of genes on the body. In this study, firstly, the 151 common DEGs were obtained by expression profile differential analysis and VENN analysis, then functional enrichment analysis was performed on them. The results of enrichment analysis showed that these DEGs were mainly enriched in infection and inflammation-related pathways and functions, such as Cytokine-cytokine receptor interaction pathway and NF-kappa B signaling pathway. Cytokines play critical roles in the pathogenesis of COVID-19 and sepsis. Much evidence suggests that cytokine storm is associated with the severity of COVID-19 patients and is a key factor in the death of COVID-19 patients (49). Studies have shown that bacteria-related molecules are recognized by Toll-like receptors of body cells and will cause a series of intracellular signaling pathways, which together activate nuclear factor κ-light-chain-enhancer of activated B cell (NF-κB), eventually leading to the expression of pro-inflammatory mediators (cytokines, chemokines, oxygen free radicals) (50). In the bioinformatics analysis of COVID-19, studies have also shown that the genes related to COVID-19 and herpes zoster are also involved in the Cytokine-cytokine receptor interaction pathway and the IL-17 signaling pathway (51). The pathway analysis results presented that these common DEGs were enriched in pathway associated with bacterial infections, staphylococcus aureus infection. Studies have shown that Staphylococcus aureus bacteremia is associated with high mortality in hospitalized patients with COVID-19 (52). In addition, these common DEGs may also be involved in certain chronic inflammatory diseases, such as Inflammatory bowel disease (IBD). Interestingly, some studies have also found that ACE2 is up-regulated in the inflamed intestinal mucosa of IBD patients, indicating that IBD patients are theoretically more susceptible to COVID-19 infection (53). However, in clinical studies, there is no data showing that the IBD population is more susceptible to COVID-19 infection, so further research is needed to determine the correlation between the two (54).

In this study, GO terms of CC indicate that these common DEGs also involve a variety of intracellular granule formation- and secretion-related pathways, including example, tertiary granule, specific granule, cytoplasmic granule lumen and cytoplasmic vesicle lumen, which have all been shown to be closely related to the function of neutrophils. It has been shown that neutrophils also play an important role in COVID-19, and their main function is phagocytosis of pathogens and debris (55). Barnes found extensive neutrophil infiltration in the pulmonary capillaries of a COVID-19 patient (56). In addition, the neutrophil-to-lymphocyte ratio (NLR) is increased in patients with COVID-19, and the neutrophil count and NLR are also the highest in critically ill patients admitted to the ICU (57). Importantly, the number and activation of neutrophils correlates with the severity of the disease (58). Furthermore, current evidence suggests that immunopathology resulting from neutrophil dysfunction is one of the important mechanisms in the pathogenesis of COVID-19 (58, 59). Typically, neutrophils can suppress and inactivate viruses through specific immune effects (release of NETs) (60, 61). Specifically, neutrophils can construct a complex network of DNA and proteins, neutrophil extracellular traps (NETs), which is a release of histone-encapsulated nucleic acid networks that retain viral particles (62). Granules, in addition to being associated with neutrophil differentiation and maturation, can also be released upon cell death and is associated with NETs (63, 64). Notably, granules embedded in NETs has been reported to have a critical pathological role in atherosclerosis, thrombosis, or tumor development (43). Data showed that circulating neutrophils exhibited an activated phenotype in COVID-19 cases and molecules associated with NETs were significantly upregulated in severe COVID-19 cases (58). In addition, Skendros discovered that complement activation enhances the platelet/NET/tissue factor/thrombin axis in COVID-19 patients (65). Nicolai noted that fibrin- and platelet-related NETs are contained in inflammatory microvascular thrombi in the kidneys, lungs, and hearts of COVID-19 patients (66). These suggest that we can disrupt the vicious cycle of thrombosis/thrombotic inflammation in COVID-19 patients by activating neutrophils and promoting the formation of NETs.

Based on the results of PPI network and hub gene extraction, *ITGAM* interacts with other genes to the strongest extent, and is probably the most important gene between COVID-19 and sepsis. In studies on COVID-19 and Guillain‐Barré syndrome, it was also found that ITGAM is an important factor in the gene regulatory network associated with the two diseases (67). ITGAM is a protective factor expressed during inflammatory injury. Some studies have found that in patients with COVID-19, the expression of *ITGAM* in females is lower than that in males, indicating that different genders have different mechanisms for regulating inflammation (68). The integrin CD11b encoded by *ITGAM* is expressed on the surface of macrophages and is involved in adhesion, migration and cell-mediated cytotoxicity (69). Studies have found that CD11b can mediate thrombus formation in COVID-19, so *ITGAM* plays an important role in thrombus formation in COVID-19 patients (70). Another study found that *ITGAM* also plays an important role in methicillin-resistant Staphylococcus aureus (MRSA)-induced sepsis. After mice were infected with MRSA, the mortality rate of ITGAM knockout mice was significantly higher than that of control mice (69). In a scoring system established with ITGAM and two other immune genes, patients with low- risk scores showed better response to immune checkpoint therapy (71). Our analysis also found significant differences in the degree of certain immune cell infiltration and immune checkpoint expression levels between COVID-19 patients with high and low *ITGAM* expression. Furthermore, our study suggested that *ITGAM* was significantly associated with some immune cells (NK cells, activated NK cells and Eosinophils) and many immune checkpoints. This also suggests that we can genotype patients with COVID-19 or patients with sepsis secondary to COVID-19 to explore which type of patients is more effective for immune checkpoint therapy.

In order to understand how common DEGs regulate COVID-19 (or sepsis) at the transcriptional level, the interactions among TFs, miRNAs and genes were investigated *via* web tools. Our results showed that the regulatory relationship between TFs (FOXC1, YY1, GATA2, PPARG and FOXL1) and genes (FCGR1B, BCL6, CD1D, MS4A4A and LTF), as well as miRNAs (hsa-mir-27a-3p, hsa-mir-26a-5p, hsa-mir-124-3p, hsa-mir-146a-5p and hsa-mir-20a-5p) and genes (*SOCS3*, *BCL6*, *CXCR4*, and *TNFSF13B*) that may play important roles in COVID-19 and sepsis. In previous bioinformatics analysis, Ahmed (72) and Islam et al. (73) both found that FOXC1, YY1, GATA2, and FOXL1 are important TFs for COVID-19. Some network pharmacology studies (74, 75) also found that PPARG may be a key therapeutic target for COVID-19. In addition, hsa-mir-27a-3p may be related to the malignant biological behavior of glioma cells (76), and may also be an important molecular feature in esophageal cancer (77). In the serum of lactating mothers with type 1 diabetes, hsa-mir-26a-5p was upregulated and was shown to be significantly associated with inflammatory responses and cytokine- and chemokine-mediated signaling pathways (78). Hsa-mir-124-3p and hsa-mir-20a-5p were also considered as potential therapeutic targets for COVID-19 in previous bioinformatics analysis (79–82). Although many previous studies have suggested that these TFs and miRNAs may have important therapeutic effects, these analytical results require further experiments to confirm their validity and authenticity.

Based on common DEGs, a gene-disease relationship network was established to understand the correlation between these genes and diseases, and these results can inspire us to develop potential drugs to treat COVID-19 with reference to the occurrence, development and treatment of these diseases. Diseases enriched by these DEGs include: liver Cirrhosis, rheumatoid arthritis, lupus erythematosus systemic and other immune and inflammation-related diseases. Recent studies have found that approximately one-third of patients with cirrhosis die within 10 days of being diagnosed with COVID-19, and two-thirds of patients with cirrhosis die before admission to the intensive care unit due to pulmonary insufficiency (83). In patients with rheumatoid arthritis, older age and comorbidities are risk factors for severe COVID-19. Glucocorticoids, appear to increase the worsening of COVID-19 outcomes (84). COVID-19 shares similarities with autoimmune diseases in clinical manifestations, immune responses, and pathogenic mechanisms. Both cause organ damage due to an excessive immune response. Autoantibodies that are hallmarks of autoimmune disease can also be detected in COVID-19 patients. Meanwhile, some COVID-19 patients have been reported to have secondary autoimmune diseases, such as Guillain-Barré syndrome or systemic lupus erythematosus (85). It appears that there are some similarities between these two diseases in terms of pathogenesis, which means that COVID-19 can be studied from this perspective.

Our drug prediction and molecular docking results suggest that emetine and progesterone can bind to multiple key targets of COVID-19 and may become new potential therapeutic drugs. Emetine is an isoquinoline alkaloid that is highly enriched in the lungs, and it has been found to have a certain inhibitory effect on the novel coronavirus in the *in vitro* environment. A real-world study showed that low-dose emetine combined with conventional antiviral drugs improved symptoms in patients with COVID-19 (86). There are also studies showing that the synergy between remdesivir and emetine can inhibit viral growth (87). Studies have found that emetine not only has a certain antiviral effect, but also can reduce the inflammatory response of patients by inhibiting the activity of NF-κB through IκBα phosphorylation, and can also reduce pulmonary hypertension by regulating various cellular processes (88). Progesterone is a sex hormone, and it also has some anti-inflammatory properties. When the novel coronavirus is infected, it can help the body control blood pressure, inhibit the formation of blood clots, and inhibit the growth of the virus. It can also regulate the body’s immune response (89).

## Conclusions

The transcriptome data of COVID-19 and sepsis versus normal controls was downloaded from public databases and then used to find DEGs for both diseases, respectively. The top 30 hub genes were screened from 151 shared DEGs. Based on these 151 DEGs, KEGG pathways and GO functions commonly involved in both diseases were explored. The results showed that they were mainly involved in infection and immune-related pathways and functions. To understand the interactions between common genes, the PPI network was delineated to show how these 151 DEGs interacted. Based on the results of hub gene extraction, ITGAM is considered to have the highest degree of interaction with other genes, and it may be potentially the most critical gene in both diseases. In order to verify our conjecture, the functional annotation and immune analysis were performed of ITGAM-related genes, and results showed that it does play a key role in immune regulation. The related genes are involved in immune-related pathways such as cytokines, anti-host transplantation disease, and infection. At the same time, it is also related to the infiltration degree of NK cells and eosinophils. In addition, there are 16 immune checkpoints associated with them, which are potential targets for the treatment of novel coronavirus and sepsis. Then these DEGs were used for screening out 8 key genes to establish an artificial neural network prediction model for COVID-19, and its AUC was as high as 0.998, indicating that the model performed very well. At the same time, a nomogram was built to predict the risk of COVID-19. To understand the role of these DEGs at the transcriptional level, TF-gene interaction network and miRNA-gene interaction network for DEGs were established to discover key TFs and miRNAs. Then the diseases most related to these DEGs were learned, mainly immune-related diseases, which suggests that we can mine effective information related to the treatment of novel coronavirus from the perspective of the development of these diseases.

## Data availability statement

Publicly available datasets were analyzed in this study. The data could be download from the GEO database of the National Center for Biotechnology Information (NCBI) (https://www.ncbi.nlm.nih.gov/geo/), accession numbers GSE147507, GSE65682 and GSE196822.

## Author contributions

FX-L conceived and designed the study. LL and LP-L provided equal contributions to research design, data analysis and article writing. RG revised the manuscript. HD, YR-S and XH-Z helped to write the manuscript. All authors contributed to the article and approved the submitted version.

## Funding

This work was supported by the Natural Science Foundation of Hunan Province (No. 2020JJ4840) and the Postgraduate Research and Innovation Project of Central South University (No. 2021zzts1093).

## Acknowledgments

The authors would like to acknowledge the GEO database for providing their platforms and those contributors for uploading their valuable datasets.

## Conflict of interest

The authors declare that the research was conducted in the absence of any commercial or financial relationships that could be construed as a potential conflict of interest.

## Publisher’s note

All claims expressed in this article are solely those of the authors and do not necessarily represent those of their affiliated organizations, or those of the publisher, the editors and the reviewers. Any product that may be evaluated in this article, or claim that may be made by its manufacturer, is not guaranteed or endorsed by the publisher.
