# Supplementary material for: Discovering common pathogenetic processes between COVID-19 and sepsis by bioinformatics and system biology approach
Source: Front Immunol. 2022 Aug 31;13:975848. doi: 10.3389/fimmu.2022.975848 (PMC9471316; doi:10.3389/fimmu.2022.975848)
Supplement: Supplementary file 1 [file Table_1.docx]

Table S1. Overview of datasets with their geo-features and their quantitative measurements in this analysis.

| Disease | GEO accession | GEO platform | Number of samples  (disease/control) | Total DEGs count | Up regulated DEGs count | Down regulated DEGs count |
| --- | --- | --- | --- | --- | --- | --- |
| COVID-19  COVID-19  Sepsis | GSE147507  GSE196822  GSE65682 | GPL18573  GPL20301  GPL13667 | 23/55  40/9  192/42 | 1855  -  1086 | 1206  -  481 | 649  -  605 |
