# Supplementary material for: Discovering common pathogenetic processes between COVID-19 and sepsis by bioinformatics and system biology approach
Source: Front Immunol. 2022 Aug 31;13:975848. doi: 10.3389/fimmu.2022.975848 (PMC9471316; doi:10.3389/fimmu.2022.975848)
Supplement: Supplementary file 2 [file Table_2.docx]

Table S2. The information of hub gene extraction from protein–protein interaction (PPI) Network.

| Gene symbol | Degree | Description |
| --- | --- | --- |
| ITGAM  FCGR3A  S100A12  FCER1G  FCGR1A  LY86  IL1RN  C3AR1  LCN2  BCL6  CAMP  RGS18  CXCR4  CLEC5A  SOCS3  CD1D  FGL2  GPR29  AQP9  CLEC4D  CD74  TNFSF13B  CD24  LTF  HCST  MPEG1  CR1  MMP8  MS4A4A  FCGR1B | 44  35  20  17  16  15  14  13  12  12  12  12  12  11  11  11  10  10  9  9  9  9  8  8  8  7  7  6  5  5 | Integrin Subunit Alpha M  Low affinity immunoglobulin gamma Fc region receptor III-A  Extracellular newly identified RAGE-binding protein  Fc fragment of IgE receptor Ig  Fc fragment of IgG receptor Ia  Lymphocyte antigen 86  Interleukin-1 receptor antagonist protein  Complement component 3a receptor 1  Lipocalin 2  B cell lymphoma 6  Cyclic adenosine monophosphate  Regulator of G-protein signaling 18  CXC motif chemokine receptor type 4  C-type lectin domain family 5, member A  Suppressor of cytokine signaling 3  Antigen-presenting glycoprotein CD1d  Fibrinogen-like protein 2  C-C motif chemokine receptor 6  Aquaporin 9  C-type lectin domain family 4, member D  CD74 molecule, major histocompatibility complex, class II invariant chain  Tumor necrosis factor (ligand) superfamily, member 13b  Cluster of differentiation 24  Lactoferrin  Hematopoietic cell signal transducer  Macrophage-expressed gene 1 protein  Complement component (3b/4b) receptor 1  Matrix metallopeptidase 8  Membrane-spanning 4-domains, subfamily A, member 4A  Fc fragment of IgG receptor Ib |
